# Valuing Insect Pollination Services with Cost of Replacement

**DOI:** 10.1371/journal.pone.0003128

**Published:** 2008-09-10

**Authors:** Mike H. Allsopp, Willem J. de Lange, Ruan Veldtman

**Affiliations:** 1 Plant Protection Research Institute, Agricultural Research Council, Pretoria, South Africa; 2 Environmental and Resource Economics Group, Council for Scientific and Industrial Research, Stellenbosch, South Africa; 3 South African National Biodiversity Institute, Kirstenbosch Research Centre, Cape Town, South Africa; University of Zurich, Switzerland

## Abstract

Value estimates of ecosystem goods and services are useful to justify the allocation of resources towards conservation, but inconclusive estimates risk unsustainable resource allocations. Here we present replacement costs as a more accurate value estimate of insect pollination as an ecosystem service, although this method could also be applied to other services. The importance of insect pollination to agriculture is unequivocal. However, whether this service is largely provided by wild pollinators (genuine ecosystem service) or managed pollinators (commercial service), and which of these requires immediate action amidst reports of pollinator decline, remains contested. If crop pollination is used to argue for biodiversity conservation, clear distinction should be made between values of managed- and wild pollination services. Current methods either under-estimate or over-estimate the pollination service value, and make use of criticised general insect and managed pollinator dependence factors. We apply the theoretical concept of ascribing a value to a service by calculating the cost to replace it, as a novel way of valuing wild and managed pollination services. Adjusted insect and managed pollinator dependence factors were used to estimate the cost of replacing insect- and managed pollination services for the Western Cape deciduous fruit industry of South Africa. Using pollen dusting and hand pollination as suitable replacements, we value pollination services significantly higher than current market prices for commercial pollination, although lower than traditional proportional estimates. The complexity associated with inclusive value estimation of pollination services required several defendable assumptions, but made estimates more inclusive than previous attempts. Consequently this study provides the basis for continued improvement in context specific pollination service value estimates.

## Introduction

Insect pollination is not only a critical ecosystem function but also an essential input in the production of a host of agricultural crops grown world-wide. Of the approximately 300 commercial crops [1] about 84% are insect pollinated [2]. Insects are responsible for 80–85% of all pollinated commercial hectares [2], with fruits, vegetables, oilseeds, legumes and fodder [3], representing approximately one-third of global food production [3]–[6], mostly pollinated by *Apis mellifera* L. (honeybees) [7]. However, the bulk of the world's staple foods are wind-pollinated, self-pollinated or propagated vegetatively, and there is a bias where values ascribed to insect pollination come from high-value per unit crops (i.e. fruits, nuts, hybrid seed, and intermediate goods for the livestock and dairy industries) [1], [3], [8]. These authors therefore argue that global food security will not be threatened if insect pollinators decline or disappear, although this ignores the diverse diet that human beings rely on [6].

Modern commercial crop production is increasingly dependent on managed pollinators (e.g. the introduction of honeybee colonies into orchards or fields to improve crop production), and less on wild insects living on the periphery of crop fields [3]. The honeybee is regarded as the most important commercial pollinator, and although other bee species are also used for commercial pollination [alkali bees (*Nomia*); mason bees (*Osmia*); leafcutter bees (*Megachile*); bumble bees (*Bombus*)], at least 90% is performed by honeybees [1]–[2], [7], [9]. Honeybees are excellent generalist pollinators, with commercial pollination being the most important derived value of commercial beekeeping worldwide [3], [10].

The “value” of managed honeybee pollination has been used to justify honey price support schemes [11]; funding for honeybee research and extension programmes [1], [12]; invasive weeds as necessary bee forage [13]–[14]; and for the preservation of indigenous vegetation [15]. In turn the “value” of the wild pollination services (pollination ecosystem service) forms part of a case for the conservation of natural biodiversity. The “global pollinator crisis” has become a *cause celebre* for those concerned with the environmental consequences of modern agriculture [4]–[6], [16]–[18]. This resulted in the International Pollinator Initiative being approved as a Convention on Biological Diversity programme. The basic premises of the initiative are that: firstly, global food security is threatened by the decline in managed honeybees and by the loss of wild pollinators; and secondly, that sustainable agriculture requires the development of alternative non-*Apis* pollinators, improvement in habitat management for wild pollinators, and improved agricultural management practices in general [1], [16]. Concerns about the global health of pollinators, and their link to food security, have been accentuated by the threat posed to honeybees by parasitic mites (*Varroa destructor* and *Acarapis woodii*) and by the as yet unexplained Colony Collapse Disorder (CCD) [19] which have seen massive honeybee losses in the USA and elsewhere.

An intense debate has developed around the respective contributions of wild and managed pollination service components, and the impact if one or the other were to be lost. Recently, Ghazoul [8] questioned the existence of a global pollinator decline, arguing that human food security is not vitally dependent on animal (mostly insect) pollination. This view was challenged by Steffan-Dewenter *et al.* [20], who cited several examples where food security has been linked to insect pollination. This debate raises the issue of whether the value of pollination as an ecosystem service can justify biodiversity conservation [5], [8]. We also question whether crop pollination by managed pollinators, mostly honeybees, can be described as an ecosystem service (e.g. [5]–[6]), and consequently, the legitimacy of advocating the preservation of wild pollinators on the basis of the derived value from managed pollinators [4], [21]. We regard managed pollination as a commercial input to agricultural crop production [22], and not an ecosystem service (see also [12]). Although crop production is regarded by some as an ecosystem service itself (e.g. [23]), this can only be true for subsistence agriculture where there are no managed inputs (i.e. fertilizers, pesticides etc.). Here we define an ecosystem service, as an ecosystem function with benefits for human life [12], [18], [24], while we define any man-made input(s) that substitute natural ecosystem services, as managed services, i.e. managed pollination services. Determining the value of crop pollination ecosystem services requires distinction between the value derived from the use of managed pollinators, and value derived from wild pollinators.

Generally, a balanced perspective on the importance of both wild and managed pollination services is lacking. For example, a recent study states that wild bees will be able to replace the pollination services provided by the domesticated honeybee, amidst the catastrophic effects of CCD (see [19]) in the USA [25]. However, although this may be true for feral honeybees in the area (also contributing to wild pollination services), no mention is made of the importance of managed honeybee pollination services in the same area. It is thus misleading to suggest wild pollination services could replace managed services based on the findings of this study.

Irrespective of this ecological debate, monetary valuation dominates natural resource conservation management decision- and policy-making. It is therefore necessary to present the importance of wild (and managed) pollinator services in monetary terms, because human decision-making is driven by financial considerations [24], [26], [27]. Unfortunately, the free market as a value estimator does a poor job (i.e. market failure) in quantifying the monetary value of ecosystem services [28]–[31]. Relative inclusiveness of market prices for ecosystem services derived benefits, are not an accurate reflection of the total value due to the difference between private and public perceived value [30], [31]. Finding a sensible alternative is therefore required for informed private and public decision-making. If the original service provided by wild pollinators is undervalued, it is likely that the cost to substitute this service with managed pollinators, will also be undervalued.

The value of wild and/or managed pollinators in commercial crop production has been estimated in many countries using different methods (see Table S1). Ascribed values have varied dramatically depending on the methodology used, with managed honeybee annual values in the USA estimated at between US$1.6 billion and US$14.6 billion (Table S1). Recently, the annual value of maintaining feral honeybee pollination ecosystem services in Australia by preventing the introduction of varroa mites, was calculated as between AUS$16.4–38.8 million (US$12.6–30.7 million) [12].

Current methods either under-estimate (by equating the value of pollination services to the direct cost of the service) [17] or over-estimate the service value (by equating the value of pollination services to the proportion of total production value dependent on insect pollination) [10], [32] (Table 1). To distinguish between the managed and wild components, we calculate the value of managed pollination as the proportion of pollination attributed to managed pollinators multiplied by the insect dependent production value. In turn, the value of wild pollination is calculated as the difference between the (total) insect dependent production- and managed pollination values [32]. Recently, the use of general insect dependence factors and the proportional allocation to managed pollinators have been criticised as being neither based on published data sources nor being regionally specific [6], [12]. This calls for a global review of the importance of insect pollination to crop production, based on experimental evidence. For example the value calculated by Cook *et al.* [12] to estimate what it would cost to substitute all feral honeybee colonies in Australia with managed ones, still assigns pollination value based on the dependence and proportion managed pollination given by Morse and Calderone [10] for the USA, based on data from McGregor [9].

**Table 1:** Current approaches to calculate pollination service value.

Approach	Formula to calculate ‘Pollination service value’	Reference
Total production value	= annual production value	
Proportion of total production value attributed to insect pollination	= annual production value x insect dependence factor*	[10], [32]
Replacement value	= (annual production value attributed to insect pollination) - (annual production value using pollinator replacement*)	Current study
Direct managed pollination value	= hive rental cost	[17]

* see Tables S2, S6 & S7.

In addition, the proportional total production value approach is flawed in several respects. Firstly, it exaggerates the economic value of pollination services because it discounts the other inputs required in the production of the commodity. A successful fruit or seed crop depends on many factors, e.g. labour and irrigation, in addition to adequate pollination [33]. Secondly, it depends on markets having an infinite elasticity of demand and is economically unrealistic [26]. That is, that increasing or decreasing production levels would have no impact on price, or on pollinator value. And finally, the proportional total production approach ignores that insect pollination is substitutable with alternatives.

A more accurate value of insect pollination services will improve conservation management decision-making capabilities, and consideration of pollination alternatives will assist crop producers. Growers might need to substitute insect pollination with alternatives for a number of reasons. These include beekeeper unwillingness to introduce their bees for pollination because of agrichemical poisoning or insufficient payment [34], the likelihood of spreading horticultural disease or bee pests [12], [19], insufficient service delivery by managed bees [7], or disappearance of wild insect pollinators [1], [6], [18]–[19].

While a number of previous studies have concerned themselves with pollination markets [12], [17], [35] only Olmstead and Wooten [36] and Southwick and Southwick [37] estimated the value of insect pollination by alternative means. These studies did not, however, pursue the nature and costs of replacements to insect pollination, and no other such studies have attempted to calculate the plausibility and cost of substituting insect in the pollination of crops. We take a different approach to valuation by estimating industry-wide replacement costs for wild and managed insect pollination services (Table 1). We adopt an approach where the value of wild and managed insect pollination services are equivalent to the amount of income lost if these components were to be replaced by alternative (non-insect) means of pollination (Table 1). Consequently the replacement cost is proposed as an estimate of the relative value of these services.

## Results

Applying traditionally used insect dependence factors, and subsequent proportional production value estimates, the total value of insect pollination services and managed pollination for the deciduous fruit industry of the Western Cape was estimated at US$358.4 and US$312.1 million respectively (Table 2). When using revised derived dependence coefficients (Table S3) and the declared number of colonies used for commercial pollination, recalculated value estimates decreased by 9% and 62% (Table 2) respectively. This is because table grapes do not require insect pollination, Morse and Calderone [10] probably overestimated insect dependencies, and managed bees are not used for apricot, peach and nectarine pollination in the Western Cape despite international recommendations to do so.

**Table 2:** Estimated annual value (US$ millions for 2005) of all insect and managed honeybee pollination to the Western Cape deciduous fruit industry (South Africa) using traditional and revised factors.

	Apples	Apricots	Peaches/Nectarines	Pears	Plums/Prunes	Grapes	Total
Total production value	208.5	12.0	48.6	113.4	35.3	83.2	501.0
***“Traditional”***							
Insect dependence factor	1.0	0.7	0.6	0.7	0.7	0.1	-
Total production value derived from insect pollination	208.5	8.4	29.2	79.4	24.7	8.3	358.5
Proportion of pollinators that are managed honeybees	0.9	0.8	0.8	0.9	0.9	0.1	
Proportion of total production value derived from insect pollination attributed to managed honeybees	187.6	6.7	23.3	71.5	22.2	0.8	312.2
***Revised estimates***							
Insect dependence factor*	0.90	0.48	0.45	0.91	0.56	0	-
Total production value derived from insect pollination	187.6	5.8	21.9	103.2	19.8	0	338.3
Number of colonies required†	37 746	3 603	7 707	42 572	20 280	0	-
Actual number used‡	15 762	30	30	8 888	21 243	0	-
Proportion of pollinators that are managed pollinators	0.418	0.008	0.004	0.209	1#	0	-
Proportion of total production value derived from insect pollination attributed to managed honeybees	78.4	0.04	0.09	21.5	19.8	0	119.8

Values were calculated using standard estimates of crop dependence on insect pollination and on the proportion of pollinators that are likely to be managed honeybees [10]; as well as using estimates of crop dependence on insect pollination from previous literature, and proportion of managed honeybee pollination derived from local industry figures.

* Factors are based on experimental evidence (Table S3).

† Number of managed honeybee colonies that need to be used for each of the deciduous fruit crops (2/ha for apples; 4/ha for pears; 1/ha for apricot cultivars; 1/ha peaches/nectarines; 6 for plums/prunes; 0 for grapes; adapted from [38] to best reflect current trends in the Western Cape).

‡ Reported use of honeybee colonies for commercial pollination in the Western Cape deciduous fruit industry ([14], personal communications from the South African Bee Industry Organisation and Deciduous Fruit Producers' Trust).

# More managed honeybee colonies are used than recommended, thus proportional contribution is at maximum.

Replacement cost estimates were used as proxies for the value of insect and managed honeybee pollination services (see Table S6 and S7; equivalent to scenario one and two described in the Materials and Method, Theoretical aspects on pollination service replacement sub-section). Pollen dusting was ineffective resulting in significantly lower total production values compared to insect pollination. Consequently using dusting as replacement for insect pollination results in values approximating traditional proportional production value estimates (Table S6) but with significantly reduced crop yield. Significant variation is evident for hand pollination value estimates. The disproportionally low fruit set figure for pears (from Table S4) resulted in higher cost estimates for hand pollination methods one and two than method three (where labour costs per hectare were assumed to be similar for all crops; Table S6). Exactly the same patterns were observed when quantifying the managed pollination service replacement value (Table S7). It should therefore be evident that pollination service value estimates are sensitive to assumptions. By subtracting the traditional and proposed replacement values of managed pollination from equivalent values calculated for all insect pollination in total, these same values could be calculated for wild pollination services (Table 3). Traditional value estimation approaches attribute significantly more value (1∶4 ratio) to managed pollination than wild pollination. In contrast, when experimental insect dependence factors and observed managed honeybee contribution were used, the ratio changed to 1∶0.4–0.6 (Table 3).

**Table 3:** Comparison of pollination service values (to the Western Cape deciduous fruit industry; US$ millions for 2005) estimated using the replacement method with those derived from traditional methods using traditional or revised factors.

Valuation method	All insect pollinators	Managed pollinators	Wild pollinators	Ratio of wild to managed value
***“Traditional”***				
Total production value approach	501.0	378.3	122.7	0.32
Proportional (dependence) production value approach	358.5	312.2	46.3	0.15
***Revised service value estimates based on experimental evidence***				
Proportional (dependence) production value approach	338.3	119.8	218.5	1.82
Production value derived from pollination services	333.9	118.0	215.9	1.83
***Cost of pollination (hive rental)***				
Current direct cost	-	1.8	-	-
Estimated direct cost assuming managed honeybee substitution	4.3	1.8	2.6	1.44
***Pollination service replacement value (income lost)***				
Pollen-dusting	292.9	107.8	185.2	1.72
Hand pollination (method 1)	161.2	44.9	116.3	2.59
Hand pollination (method 2)	433.8	122.8	310.9	2.53
Hand pollination (method 3)	77.0	28.0	49.1	1.75

In summary, depending on which of the four value estimation methods were used, replacement values varied significantly due to differences in pollination efficiencies and costs of the different replacement methods, and varied between 0.23–1.30 of proportional total production estimates. However, irrespective of the choice of replacement method, wild pollination services have been under estimated in the past.

## Discussion

The Peoples' Republic of China is by far the biggest deciduous fruit producing country in the world [22]. South Africa can be regarded as a small role player with global contributions varying between 0.8% (plums) and 3% (apricots) from approximately 74 716 hectares under cultivation in 2005 [22]. The vast majority of this area (80.3%) is in the Western Cape with a total annual production value estimated at US$501 million (see Table S2) in 2005 of which apples and pears contributed 42% and 23% respectively. Given the relative importance of pollination for the deciduous fruit industry there is a clear need to better estimate the value of such services and the revised calculations presented are considered to be an accurate reflection of the value of insect and managed honeybee pollination in the Western Cape. It is clear that while the traditional insect dependence factors (e.g. [10]) over-estimate the importance of insect pollination, the same cannot be said of the relative importance given to managed and wild pollinators, with the latter being underestimated. The declared use of pollination units (number of managed hives used during season) in deciduous fruit orchards is only 41% of what is recommended [12], [38], and reflects either that not as many pollinators are required as internationally recommended stocking rates would indicate, and/or that additional pollination is provided by wild pollinators. Possible reasons for this may be higher numbers of wild honeybees in South Africa compared with other parts of the world [39]; significant numbers of “residential” honeybee colonies (hived but unmanaged) that remain permanently on farms; intensive crop production areas may have spill-over effects between orchards; and finally that some insect pollination requirements have been exaggerated.

Whatever the reasons for the discrepancy, the valuation method that is used, or the relative contributions of managed and wild pollinators, it is apparent that both services are grossly undervalued. The contribution of managed honeybee pollination is found to be between US$28.0–122.8 million, for which only US$1.8 million is presently being paid. Similarly, the contribution of wild pollinators is found to be between US$49.1–310.9 million for which there is no direct payment from producers. These values illustrate the importance of maintaining natural and other forage areas for the conservation of insect pollinators; thereby informing government initiatives on land use management. For example, various alien *Eucalyptus* spp. which are important summer forage for managed bees in the Western Cape are listed for removal (Conservation of Agricultural Resources Act, 1983). Although alien removal has benefits (including employment creation and alleviation of the current water shortages [40]), removal could negatively affect the managed bee industry. These positive externalities of aliens must be accounted for to achieve a balanced view on natural resource management and conservation strategies.

Using the replacement approach it is evident that the higher the relative level of efficiency, the smaller the associated income loss and consequently the lower the value of insect pollination services. In this study, hand pollination replacement calculations according to methods one and three proved more effective than pollen dusting and hand pollination method two. The range of value estimates presented here are significantly higher than current market prices for pollination services, although lower than traditional proportional estimates. This market failure (i.e. where the market price of the pollination service does not reflect its true value) could promote unsustainable landscape use since both managed and wild pollinators are dependent on natural and semi-natural landscapes as forage, particularly during off-season. If managers are forced to replace insect pollination at an industry scale, an increase in the input cost structure of the deciduous fruit industry is expected. This could have negative impacts on the profitability and international competitiveness of this industry in the medium term.

The substantial variation in the value estimation of insect pollination services, depending on the method used, reflects the pre-dominance of the assumptions used and the paucity of relevant and accurate data, and supports the call [6], [12] for a global review of the insect dependence of commercial crops. The assumption that hand pollination will not improve the crop yield achieved with insect pollination, while pollen dusting results in a significantly lower yield is crucial in our replacement calculations. Should these assumptions be inaccurate, or change over time, the viability of the replacement methods will also change. In some crops hand pollination might increase fruit set and crop yield, in comparison to insect pollination, and hand pollination in these crops might be a viable proposition even if insect pollinators are not a limiting factor (i.e. mango or custard apples [41], [42]). Among the deciduous fruit crops, the yield of pears is the most likely to be able to be significantly improved by hand pollination with other deciduous crops already at their physiological maximum [43]. The economic feasibility of hand pollination will hence need to be considered on an individual crop basis, depending on the improved yield that can be obtained against the added costs of hand pollination. Under insect pollination limitation it would also be beneficial for crop producers to improve the efficiency of alternative pollination methods. This might entail the selection of more parthenocarpic cultivars, or self-fruitful cultivars, or an improvement in pollen dusting efficiency.

Estimating a fully inclusive value for insect (wild or managed) pollination services is challenging. If such estimates are based on values derived from commercial crops alone, the total pollination service value will constantly be underestimated because of externalities (such as incidental pollination of pastures, gardens and natural vegetation that need to be included in the value estimations). The value of pollination services derived from insects is only one of many in the total value chain of insects. If this chain could be valued, better and more inclusive resource management decision-making will follow. However, data limitations are likely to be the constraining factor for most other species. This problem provides strong support for a precautionary approach when managing most species. Tangible and scientifically defendable value estimates reduce reliance on arbitrary judgement, and emphasize the fact that ecosystem goods and services are certainly not ‘free’. In doing so, the credibility of public buy-in in biodiversity conservation, will be promoted, making it easier for policymakers to defend conservation investment against competing government expenditure.

## Materials and Methods

The Western Cape deciduous fruit industry (South Africa) was used as a case study to assess the value of wild and managed pollination services. This industry was chosen because of its well documented dependence on managed honeybees [14]. However, its dependence on wild pollinators has not yet been reported. Here we present the contribution (in terms of production value) of each wild and managed service for the first time, as well as their replacement cost.

For comparison with previous studies, we first calculate the value of all insect and managed pollination services using the traditional proportional total production approach and the dependency coefficients of Morse and Calderone [10], and industry data [22] (Table S2). We then re-calculate these data using revised insect dependence coefficients. It is difficult to ascribe to any single crop a value for insect pollination dependence, because of the extreme variation between cultivars in any given crop, and because the pollination dependence of most commercial cultivars is not well known [43]. Many cultivars are completely self-unfruitful and can only produce fruit after cross pollination, requiring an insect vector, as wind pollination is insignificant in deciduous fruit crops [7]. Other cultivars are, however, self-fruitful, or partially self-fruitful, and can produce some fruit when pollinated by their own pollen. Cultivars may also vary in self-fruitfulness from year to year, and from location to location, and with age [7]. Some cultivars are also partially parthenocarpic and can set fruit without pollination or fertilization. The variability is such that some plum cultivars are self-sterile, relying completely on insect pollination, while others are totally self-fertile [43]. As a general rule, apples, plums and pears are self-unfruitful and need insect pollinators while apricots, peaches and nectarines are self-fruitful, although insect pollination often improves fruit set and fruit quality [7]. The pollination of grapes is often viewed as a “vexed” question [44], with almost no studies and data. Most reports conclude that the cultivated grape (*Vinis vinifera*) is pollinated by wind and are rarely visited by insects [7], [9], [44], [45].

### Revised insect dependence factors

Precise data on the insect dependence of the deciduous fruit crop in the Western Cape is not available, and would require an assessment of each planted cultivar. Nonetheless, more data are available than have generally been used in earlier estimates [7], [9], [10] and these data have been used to calculate revised insect dependence factors. Most of these data come from controls used in cultivar compatibility trials, and not in experiments to determine insect dependence, and in many cases insect dependence factors have been assigned based on published raw data. From the various reports of insect dependence for the deciduous fruit cultivars, the most appropriate factor is selected (Supplementary Table S3).

In addition to the proportional insect contribution being re-calculated using revised insect dependence factors, the relative contribution of managed and wild pollinators is also re-calculated. The pollination requirements of the deciduous fruit industry in the Western Cape is determined by the hectarage requiring pollination [22] and the recommended pollination stocking rates per hectare for each crop [38]. These recommendations are in accordance with international recommended stocking rates [7], [12]. We compare these data with beekeeper reports on the actual usage of managed honeybee colonies for deciduous fruit pollination in the Western Cape ([14], personal communications from the South African Bee Industry Organisation and Deciduous Fruit Producers' Trust) allowing for accurate proportional allocation of the value of each crop to managed and wild pollination.

### Theoretical aspects on pollination service replacement

In considering the replacement of pollinators, we considered two hypothetical scenarios. The first scenario assumes that no insects (wild or managed) remain for pollination. This may be due to increased hectares under cultivation and concurrent destruction of natural ecosystems, as well as biodiversity loss due to climate change, and insinuates a global pollinator crisis [1], [3], [5]–[6], [18]. If true, this will require all insect pollination to be replaced. Our second scenario assumes that managed pollination is not commercially viable or possible, with only its contribution to be replaced (wild pollination service remains).

Possible options for the replacement of pollination services are limited. The use of managed non-honeybee pollinators is not considered feasible in the Western Cape. Non-*Apis* pollinators have been commercially used in many parts of the world [1]–[2], [7] but never in South Africa. In any event, should there be a global pollinator crisis based on decreasing forage or nest-sites, it is unlikely that non-*Apis* species will fare any better than will honeybees, and hence unlikely that they will be viable alternatives.

A second alternative is for a grower to convert his crop to parthenocarpy [46], the production of fruit without fertilization, which is found in the commercial production of numerous crops and can be induced by various methods. Parthenocarpy cannot be induced, however, in many crop types and cultivars, and will not be a practical short-term solution should insect pollination no longer be available.

The only viable alternative to insect pollination is considered to be pollination by mechanical means. This requires pollen to be collected from appropriate cross-pollinating cultivars, for hand or mechanical delivery to the target crop. Pollen is collected by hand at the popcorn stage, and the pollen mechanically separated, dried and stored [47]. Once collected and prepared, the pollen is delivered to the target blossoms manually (hand pollination) or mechanically (pollen dusting). Pollen dusting may be done by aircraft and helicopters (efficacy unverified) or with hand operated pollen blowers [48]. A number of commercial companies promote the use of pollen dusters (e.g. [49]) with reliable pollination efficacy data on this method being available [47], [50]. Two applications of pollen are typically recommended for commercial pollen dusting. Hand pollination entails the manual application of pollen to the stigmas of individual flowers by means of a paint brush or something similar [42], [51], [52]. It is best to pollinate closed blossoms at the balloon stage by pushing the paintbrush or pencil into the flower and twisting it around [42], so that pollen can be delivered to more than one stigma, so increasing the crop set and quality [52]. An orchard will typically need to be hand pollinated at least twice, so that sufficient flowers at the correct stage are available and can be pollinated.

### Calculating pollination service replacement

In considering pollen dusting and hand pollination as the only viable replacements of insect pollination services, we need to take into account the relative efficacy of the methods. Pollen dusting is expected to be less effective than normal insect pollination, both in terms of fruit set and fruit quality [50]. Fruit set resulting from pollen dusting is estimated to be 73.5% less as compared to insect pollination (an average of the results of [47], [48]. Fruit weight from pollen dusting is estimated to be 42% less when compared to insect pollination [53]. By contrast hand pollination of flowers is expected to deliver equal or more fruit set than insect pollination [42], and to deliver as big or bigger fruit [42], [51], [54]. For any crop, or cultivar, however, a point will be reached where hand pollination cannot increase yield or quality, and any hand pollination advantages are compromised by resource limitations. This is expected to be the case in most deciduous fruit cultivars and hand pollination is therefore assumed to deliver an equivalent crop to insect pollination.

Additionally, data on the percentage fruit set resulting from hand pollination is required to calculate labour costs for the hand pollination of the numbers of flowers that need to be pollinated to yield the same number of fruit as produced in the orchard by insect pollination. As in the case of insect dependence, these figures are difficult to determine because of the extreme variability between cultivars. This is complicated by a compatibility issue, with each cultivar pair having a specific fruit set percentage. As an example of this variability, pairs of cultivars in apricots have fruit set between 0.4% and 54% [55]. As with the estimation of the insect dependence coefficient, a truly accurate regional figure can only be determined by investigating all cultivar pairs grown in the region. As this is clearly impractical, the most appropriate fruit set percentage is selected from the various reports of cultivar dependence for the deciduous fruit cultivars (Table S4).

The replacement value of insect and managed honeybee pollination was estimated using pollen dusting (standard method) and three hand pollination methods (all pollen prices and application costs are deflated to 2005 values). Hand pollination methods one and two are based on the expected number of flowers that are needed to be pollinated. Method one is based on the number of flowers that will need to be hand pollinated to produce the equivalent amount of fruit as are produced during insect pollination, and estimates that it will take five seconds to pollinate each flower. Method two assumes that the pollination of a single flower takes twice as long as the picking of a fruit, a conservative assumption. If a fifty percent set is obtained from hand pollinating fruit for a particular crop, this means that twice as many flowers need to be pollinated than fruit picked. Therefore, for an orchard of this crop, the labour cost of pollinating flowers is four times the cost of picking the fruit. As the average harvest labour cost in the deciduous fruit industry in South Africa in 2005 is known, for each deciduous fruit crop [22], the labour component can be determined. These costs can be re-calculated depending on the expected set for hand pollination for each deciduous fruit crop. Method three uses the same pollen costs as methods one and two, but estimates labour costs from published accounts of hand pollination of deciduous fruit trees (Table S5). The 180 man-days per hectare required for the hand pollination of apples is assumed to be a good proxy of other deciduous fruit types for which equivalent published information is not available.

The following additional assumptions were made in this replacement value estimation:

i) A long term average (1996–2007) ZAR/US$ exchange rate of 6.74388∶1 was used.
ii) We used 2005 as our base year, and all prices were discounted accordingly.
iii) It would not be cost-effective for farmers to produce their own pollen for cross pollination if compared to commercial pollen available at US$175.7 per hectare for hand pollination and US$234.1 per hectare for pollen dusting (see [49], based on US prices and a ZAR/US$ exchange rate of 7.1). It is assumed that pollen could be produced commercially at the same cost in South Africa as currently offered internationally.
iv) The rental cost of commercial honeybee colonies for pollination in 2005 was US$38.8, and the number of colonies applied per hectare is as recommended (2/ha for apples; 4/ha for pears; 1/ha for apricot cultivars; 1/ha peaches/nectarines; 6 for plums; 0 for grapes (adapted from [38] to best reflect current trends in the Western Cape).
v) Pollen dusting requires two applications per hectare. Each application takes approximately half a day to complete for a single operator.
vi) General labour cost in 2005 is estimated at US$12.1 per day per person.

Finally, after calculating the four replacement estimates for both the total insect loss and managed honey bee loss scenarios, the value of wild pollination services is calculated by subtracting the value obtained in the second scenario from the value obtained in the first. Strictly speaking, because wild pollinators may interact with managed pollinators thereby increasing crop pollination [6], [56]–[58], the value of wild pollination as calculated above represents both wild pollination in the absence of managed pollination and interactive effects when both these pollination services are present. However, seeing as such interactive effects still require the presence of wild pollinators, and hence an ecosystem service, we do not distinguish here between these two sub components. Total insect, managed and wild pollination values using the replacement method are subsequently compared to those derived from traditional factors and methods, as well as traditional methods but with revised factors.

## Supporting Information

Table S1 Biogeographic-specific estimated value of managed bee pollination for commercial crops. Value estimates are first in biogeographical order and then chronology. The “proportion” of agricultural produce refers to the portion of crop value that can be attributed to managed bees for pollination (as opposed the remaining portion that is attributable to wild insect pollination).
(0.05 MB DOC)

Table S2 Summary statistics for the deciduous fruit industry in South Africa for 2004-2005 season. The following values from the deciduous fruit industry [1] were used to calculate replacement estimates.
(0.06 MB DOC)

Table S3 Insect dependence of deciduous fruit crops. The re-assessment of dependency of deciduous fruit upon insects is based on data where trees were “quarantined” with cages or bags to exclude pollination of insects. The fruit set or yield in the “quarantined” trees were compared with that obtained in the open field and normal insect-pollinated conditions. Fruit production in the cages or bags results from self-pollination, wind pollination or parthenocarpy, and is not insect-mediated. Yield was use for cases where both yield and set data is available.
(0.05 MB DOC)

Table S4 Fruit set percentage from hand pollination of deciduous fruit crops. Previous studies using hand pollination and insect exclusion treatments were used to estimate crop specific fruit set. The rationale for selected fruit set value is provided.
(0.04 MB DOC)

Table S5 Time required for one person to hand pollinate apple and pear fruit trees.
(0.04 MB DOC)

Table S6 Insect pollination replacement costs for the Western Cape deciduous fruit industry.
(0.07 MB DOC)

Table S7 Managed honeybee replacement cost for the Western Cape deciduous fruit industry. Identical to Table S6, except for the use of observed instead of estimated number of honeybee colonies.
(0.07 MB DOC)
