# Supplementary material for: Valuing Insect Pollination Services with Cost of Replacement
Source: PLoS One. 2008 Sep 10;3(9):e3128. doi: 10.1371/journal.pone.0003128 (PMC2519790; doi:10.1371/journal.pone.0003128)
Supplement: Table S1 — Biogeographic-specific estimated value of managed bee pollination for commercial crops. Value estimates are first in biogeographical order and then chronology. The “proportion” of agricultural produce refers to the portion of crop value that can be attributed to managed bees for pollination (as opposed the remaining portion that is attributable to wild insect pollination). (0.05 MB DOC) [file pone.0003128.s001.doc]

**Table S1.** Biogeographic-specific estimated value of managed bee pollination for commercial crops.

| Reference | Country | Value | Method (as described by authors) |
| --- | --- | --- | --- |
| 1 | USA | US$ 40.0 billion | Proportion of fruit, nuts, vegetable seed, oilseed, fodder and value derived from fodder due to managed bee pollination. |
| 2 | USA | US$ 18.9 billion | Proportion of fruit, nuts, vegetable seed, oilseed, fodder and value derived from fodder due to managed bee pollination. |
| 3 | USA | US$ 4.6 billion | Proportion of fruit, nuts, vegetable seed, fodder seed and oilseed due to insect pollination. |
| 4 | USA | US$ 0.18-3.0 billion | Proportion of some fruit, nuts, vegetable seed, fodder seed and oilseed due to insect pollination, incorporating yield fluctuations. |
| 5 | USA | US$ 9.3 billion | Proportion of fruit, nuts, vegetable seed, fodder seed and oilseed due to managed bee pollination. |
| 6 | USA | US$ 1.6-5.7 billion | Values based on decreasing commodity costs resulting from improved yield due to commercial pollination. |
| 7 | USA | US$ 14.6 billion | Proportion of fruit, nuts, vegetable seed, fodder seed and oilseed due to managed bee pollination. |
| 8 | Canada | US$ 0.66 billion (Can$ 782 million) | Proportion of fruit, nuts, vegetable seed, fodder seed and oilseed due to managed bee pollination. |
| 9 | Russia | US$ 0.08 billion  (2.2 billion roubles) | Additional yield in all insect pollinated crops resulting from managed bee pollination |
| 10 | EU | US$ 6.4 billion  (5 billion ecus) | Proportion of fruit, nuts, vegetable seed, fodder seed and oilseed due to insect pollination. |
| 11 | UK | US$ 0.38 billion (£202 million) | Proportion of fruit, nuts, vegetable seed, fodder seed and oilseed due to managed bee pollination. |
| 12 | Australia | US$ 0.5-0.9 billion (AUS$ 0.6-1.2 billion) | Values based on decreasing commodity costs resulting from improved yield due to commercial pollination. |
| 13 | New Zealand | US$ 1.54 billion (NZ$ 2.25 billion) | Total market value of all insect pollinated crops |
| 14 | South Africa | US$ 0.61 billion (ZAR 4.1 billion) | Proportion of fruit, nuts, vegetable seed, fodder seed and oilseed due to managed bee pollination. |

Value estimates are first in biogeographical order and then chronology. The ‘proportion’ of agricultural produce refers to the portion of crop value that can be attributed to managed bees for pollination (as opposed the remaining portion that is attributable to wild insect pollination).

1. Martin EC (1975) The use of bees for crop pollination. In: Dadant & Sons, editors. The hive and the honeybee. Hamilton (U S A): Dadant & Sons Incorporated. pp. 579-614.
2. Levin MD (1984) Value of bee pollination to United States Agriculture. Am Bee J 124: 184-186.
3. O’Grady JH (1987) Market failure in the provision of honey bee pollination: A heuristic investigation [dissertation]. Vermont (U S A): University of Vermont.
4. Olmstead AL, Wooten D (1987) Bee pollination and productivity growth: The case of alfalfa. Am J Agri Econ 69: 56-63.
5. Robinson WS, Nowogrodzki R, Morse RA (1989) The value of honey bees as pollinators of the United States crops. Am Bee J 7: 477-487.
6. Southwick EE, Southwick L (1992) Estimating the economic value of honey bees (Hymenoptera: Apidae) as agricultural pollinators in the United States. J Econ Entomol 85: 621-633.
7. Morse RA, Calderone NW (2000) The value of honey bees as pollinators of U.S. crops in 2000. Bee Cult 128: 1-15.
8. Winston ML, Scott CC (1984) The value of bee pollination to Canadian agriculture. Can Beekeeper 11: 134.
9. Soldatov VI (1976) Economic effectiveness of bees as pollinators of agricultural crops. In: Kozin RB, editor. Pollination of entomophilous agricultural crops by bees. New Delhi (India): Amerind Publishing Co. pp. 125-134.
10. Borneck R, Merle B (1989) Trial to evaluate the economical incidence of the pollinating honeybee in the European Agriculture. Apiacta 24: 33-38.
11. Carreck N, Williams I (1998) The economic value of bees in the UK. Bee World 79: 1115-1123.
12. Gill RA (1991) The value of honeybee pollination to society. Apiacta 26: 97-105.
13. Matheson A, Schrader M (1987) The value of bees to New Zealand’s primary production. Nelson (New Zealand): Ministry of Agriculture and Fisheries. 5 p.
14. Allsopp MH (2004). Cape honeybee (*Apis mellifera capensis* Eschscholtz) and varroa mite (*Varroa destructor* Anderson & Trueman) threats to honeybees and beekeeping in Africa. Intern J Tropi Insect Sci 24: 87-94.
