# Supplementary material for: Valuing Insect Pollination Services with Cost of Replacement
Source: PLoS One. 2008 Sep 10;3(9):e3128. doi: 10.1371/journal.pone.0003128 (PMC2519790; doi:10.1371/journal.pone.0003128)
Supplement: Table S2 — Summary statistics for the deciduous fruit industry in South Africa for 2004-2005 season. The following values from the deciduous fruit industry [1] were used to calculate replacement estimates. (0.06 MB DOC) [file pone.0003128.s002.doc]

**Table S2:** Summary statistics for the deciduous fruit industry in South Africa for 2004-2005 season.

|  | Apples | Apricots | Peaches / Nectarines | Pears | Plums | Table Grapes |
| --- | --- | --- | --- | --- | --- | --- |
| Production (tonnes per year) | 658 940 | 82 282 | 184 783 | 328 631 | 55 278 | 351 483 |
| Total Hectares | 20 774 | 4 302 | 10 492 | 11 812 | 4 581 | 22 755 |
| Total Hectares (W Cape) | 20 311 | 4 224 | 9 551 | 11 808 | 4 370 | 9 818 |
| Production Hectares (age > 3 years) | 19 303 | 3 667 | 8 466 | 10 647 | 3 543 | 19 211 |
| Production Hectares in W Cape (age > 3 years) | 18 873 | 3 603 | 7 707 | 10 643 | 3 380 | 8 289 |
| Total production value (ZAR millions)* | 1 438 | 82 | 360 | 765 | 250 | 1 299 |
| Total value of production in W Cape (ZAR millions)* | 1 406 | 81 | 328 | 765 | 238 | 561 |
| Yield (tonnes per hectare) | 55 | 20 | 25 | 45 | 25 | 20 |
| Hectares requiring pollination | 18 873 | 3 603 | 7 707 | 10 643 | 3 380 | 0 |
| Trees per hectare (standard spacing) | 1 650 | 1 250 | 1 600 | 1 650 | 1 425 | 1 667 |
| Yield (kilograms per tree) | 33.3 | 16.0 | 15.6 | 27.3 | 17.5 | 12.0 |
| Average fruit weight (grams) | 142 | 45 | 100 | 139 | 62 | - |
| Number of fruit harvested per tree | 235 | 356 | 156 | 196 | 283 | - |
| Harvesting labour costs | 5 670 | 15 912 | 17 025 | 4 635 | 21 657 | 5 279 |
| Production cost per hectare | 111 387 | 84 735 | 110 888 | 95 524 | 101 043 | 125 528 |

The following values from the deciduous fruit industry [1] were used to calculate replacement estimates.

*ZAR can be converted to US$ by dividing by 6.74388

1. Deciduous Fruit Producers Trust (DFPT) (2005) Key Deciduous Fruit Statistics, 2005. Paarl (South Africa): Optimal Agricultural Business Systems. 80 p.
