# Supplementary material for: Valuing Insect Pollination Services with Cost of Replacement
Source: PLoS One. 2008 Sep 10;3(9):e3128. doi: 10.1371/journal.pone.0003128 (PMC2519790; doi:10.1371/journal.pone.0003128)
Supplement: Table S3 — Insect dependence of deciduous fruit crops. The re-assessment of dependency of deciduous fruit upon insects is based on data where trees were “quarantined” with cages or bags to exclude pollination of insects. The fruit set or yield in the “quarantined” trees were compared with that obtained in the open field and normal insect-pollinated conditions. Fruit production in the cages or bags results from self-pollination, wind pollination or parthenocarpy, and is not insect-mediated. Yield was use for cases where both yield and set data is available. (0.05 MB DOC) [file pone.0003128.s003.doc]

**Table S3:** Insect dependence of deciduous fruit crops.

| Crop | Insect Dependence Factor [Reference] | Factor selected | Rationale |
| --- | --- | --- | --- |
| Apple | 0.92 [1]; 0.91 [2]; 0.96; 1.00 [3]; 0.90 [4]; 0.00; 0.65 [5]; 0.97 [6]; 0.96 [7]; 0.63 [8]; 0.90 [9]; 0.70 [10] | 0.90 | Soltész [9] examined 292 apple cultivars, by far the most comprehensive study |
| Pears | 0.96 [1]; 0.33 [11]; 0.91 [12]; 0.92 [13]; -0.41 [14]; 0.49 [15] | 0.91 | Nyéki *et al.* [12] is the most comprehensive study, using 14 pear cultivars |
| Plums & Prunes | 0.62 [16]; 0.29 [7]; 0.78 [17] | 0.56 | Averaged |
| Peaches & Nectarines | 0.44; 0.54 [1]; 0.61 [18]; 0.45 [19]; 0.43 [20] | 0.45 | Nyéki *et al.* [19] is the most comprehensive, with data from 66 varieties over 36 years |
| Apricots | 0.32 [21]; 0.47 [22]; 0.66 [23] | 0.48 | Averaged |
| Grapes | 0.00 [24]; 0.00 [25]; 0.00 [26] | 0.00 | - |

The re-assessment of dependency of deciduous fruit upon insects is based on data where trees were “quarantined” with cages or bags to exclude pollination of insects. The fruit set or yield in the “quarantined” trees were compared with that obtained in the open field and normal insect-pollinated conditions. Fruit production in the cages or bags results from self-pollination, wind pollination or parthenocarpy, and is not insect-mediated. Yield was use for cases where both yield and set data is available.

1. Bulatovic S, Konstantinovic B (1960) The role of bees in the pollination of the more important kinds of fruit in Serbia. In: Mittler TE, editor. Proceedings of the First International Symposium on Pollination. Lindhska (Sweden): Lindhska Press. pp. 167-172.
2. Griggs WH, Iwakiri BT (1960) Orchard tests of beehive pollen dispensers for cross-pollination of almonds, sweet cherries and apples. Proc Am Soc Hort Sci 75: 114-128.
3. Free JB (1964) Comparison of the importance of insect and wind pollination of apple trees. Nature 201: 726-727.
4. Free JB (1966) The pollinating efficiency of honey-bee visits to apple flowers. J Hort Sci 41: 91-94.
5. Free JB, Smith BD, Stott KG, Williams IH (1974) The pollination of self-fertile apple trees. J Hort Sci 49: 301-304.
6. Anderson RH (1980) Pollination of apples and pears. Elgin (South Africa): Elgin Co-operative Fruitgrowers. 44 p.
7. Benedek P, Nyéki J (1996) Fruit set of selected self-sterile and self-fertile fruit cultivars as affected by the duration of insect pollination. Acta Hort 423: 57-63.
8. de Witte K, Vercammen J, van Daele G, Keulemans J (1996) Fruit set, seed set and fruit weight in apple as influenced by emasculation, self-pollination and cross-pollination. Acta Hort 423: 177-183.
9. Soltész M (1997) Pollination and fertilization of apple cultivars in Hungary. Acta Hort 437: 445-448.
10. Khan MR, Khan MR (2004) The role of honeybees *Apis mellifera* L. (Hymenoptera: Apidae) in pollination of apple. Pakistan J Biol Sci 7: 359-362.
11. Langridge DF, Jenkins PT (1972) A study on pollination of Packham’s Triumph pears. Aust J Exp Agric 12: 328-330.
12. Nyéki J, Göndörné Pintér M, Szabó Z (1994) Recent data on fertilization of pear varieties. Acta Hort 367: 87-91.
13. van den Eijnde J (1996) Pollination of pear by bumblebees (*Bombus terrestris* L.) and honeybees (*Apis mellifera* L.). Acta Hort 423: 73-78.
14. Sharifani MM, Jackson JF (2001) Influence of caging on pollination and fruit set of two pear cultivars. Acta Hort 561: 235-241.
15. Maccagnani B, Ladurner E, Santi F, Burgio G (2003) *Osmia cornuta* (Hymenoptera, Megachilidae) as a pollinator of pear (*Pyrus communis*): fruit- and seed-set. Apidologie 34: 207-216.
16. Langridge DF, Goodman RD (1985) Honeybee pollination of Japanese plums (*Prunus salicina* Lindl. cv. Satsuma) in the Goulburn Valley, Victoria. Aust J Exp Agric 25: 227-230.
17. Szabó Z (2003). Plum (*Prunus domestica* L.). In: Kozma P, Nyéki J, Soltész M, Szabó Z, editors. Floral Biology, Pollination and Fertilisation in Temperate Zone Fruit Species and Grape. Budapest: Akamémiai kaidó. pp. 383-410.
18. Langridge DF, Jenkins PT, Goodman RD (1977) A study on pollination of desert peaches cv. Crawford. Aust J Exp Agric 17: 697-699.
19. Nyéki J, Szabó Z, Soltész M, Kovács J (1998) Open pollination and autogamy of peach and nectarine varieties. Acta Hort 465: 279-284.
20. Szabó Z, Nyéki J, Soltész M (2003). Peaches (*Prunus persica* L. Batsch). In: Kozma P, Nyéki J, Soltész M, Szabó Z, editors. Floral Biology, Pollination and Fertilisation in Temperate Zone Fruit Species and Grape. Budapest: Akamémiai kaidó. pp. 425-434.
21. Langridge DF, Goodman RD (1981) Honeybee pollination of the apricot cv. Trevatt. Aust J Exp Agric Anim Husb 21: 241-244.
22. McLaren GF, Fraser JA (1996) Pollination compatibility of ‘Sundrop’ apricot and its progeny in the ‘Clutha’ series. N Z J Crop Hort Sci 24: 47-53.
23. Szabó Z, Nyéki J, Andrásfalvy A (1999) Evaluation of some Romanian apricot varieties in Hungary, Acta Hort 488: 211-214.
24. Mullins MG, Bouquet A, Williams LE (1992) Biology of the Grapevine. Cambridge (United Kingdom): Cambridge University Press. 239 p.
25. Kozma P (2003) Exploration of flower types in grapes (Vitis vinifera). In: Kozma P, Nyéki J, Soltész M, Szabó Z, editors. Floral Biology, Pollination and Fertilisation in Temperate Zone Fruit Species and Grape. Budapest: Akamémiai kaidó. pp. 75-225.
26. Heazlewood JE, Wilson S (2004) Anthesis, pollination and fruitset in Pinot Noir. Vitis 43: 65-68.
