# Supplementary material for: Valuing Insect Pollination Services with Cost of Replacement
Source: PLoS One. 2008 Sep 10;3(9):e3128. doi: 10.1371/journal.pone.0003128 (PMC2519790; doi:10.1371/journal.pone.0003128)
Supplement: Table S4 — Fruit set percentage from hand pollination of deciduous fruit crops. Previous studies using hand pollination and insect exclusion treatments were used to estimate crop specific fruit set. The rationale for selected fruit set value is provided. (0.04 MB DOC) [file pone.0003128.s004.doc]

**Table S4.** Fruit set percentage from hand pollination of deciduous fruit crops.

| Crop | Fruit set percentage from hand pollination [Reference] | Percentage selected | Rationale |
| --- | --- | --- | --- |
| Apple | 37 [1]; 14 [2]; 70 [3]; 15 [4]; 52 [5]; 36 [6]; 50 [7]; 51 [8]; 63 [9]; 21 [10]; 56 [11]; 57 [12] | 50 | Median, and is close to the 0.57 found by [12] is the most comprehensive study, using 77 cultivar combinations. |
| Apricots | 54 [13]; 25 [14] | 25 | [14] is based on 62 cultivar combinations, and is the most comprehensive study. |
| Peaches & Nectarines | No data | 25 | The apricot data is considered to be the most likely to be accurate for peaches and nectarines. |
| Pears | 8 [15]; 4 [16] | 6 | Averaged |
| Plums | No data | 25 | The apricot data is considered to be the most likely to be accurate for plums. |

Previous studies using hand pollination and insect exclusion treatments were used to estimate crop specific fruit set. The rationale for selected fruit set value is provided.

1. Griggs WH, Iwakiri BT (1960) Orchard tests of beehive pollen dispensers for cross-pollination of almonds, sweet cherries and apples. Proc Am Soc Hort Sci 75: 114-128.
2. Free JB (1964) Comparison of the importance of insect and wind pollination of apple trees. Nature 201: 726-727.
3. Anderson RH (1980) Pollination of apples and pears. Elgin (South Africa): Elgin Co-operative Fruitgrowers. 44 p.
4. Rejman A (1983) The influence of pollinators on fruit set and some characteristics of Close apples. Acta Hort 139: 29-31.
5. Wertheim SJ (1991) *Malus* cv. Baskatong as an indicator of pollen spread in intensive apple orchards. J Hort Sci 66: 635-642.
6. de Putter H, Kemp H, de Jager A (1996) Influence of pollinizer on fruit characteristic of apple. Acta Hort 423: 211-217.
7. de Witte K, Vercammen J, van Daele G, Keulemans J (1996) Fruit set, seed set and fruit weight in apple as influenced by emasculation, self-pollination and cross-pollination. Acta Hort 423: 177-183.
8. Keulemans J, Bruselle A, Eyssen R, Vercammen J, van Daele, G (1996) Fruit weight in apple as influenced by seed number and pollinizer. Acta Hort 423: 201-206.
9. Volz RD, Tustin DS, Ferguson IB (1996) Pollination effects on fruit mineral composition, seeds and cropping characteristics of ‘Braeburn’ apple trees. Scientia Hort 66: 169-180.
10. Kron P, Husband BC, Kevan PG, Belaoussoff S (2001) Factors affecting pollen dispersal in high-density apple orchards. Hort Sci 36: 1039-1046.
11. Sheffield CS, Smith RF, Kevan PG (2005) Perfect syncarpy in apple (*Malus* x *domestica* ‘Summer McIntosh’) and its implications for pollination, seed distribution and fruit production (Rosaceae: Maloideae). Annals Bot 95: 583-591.
12. Matsumoto S, Egushi T, Bessho H, Abe K (2007) Determination and confirmation of S-RNase genotypes of apple pollinators and cultivars. J Horti Sci Biotech 82: 323-329.
13. McLaren GF, Fraser JA (1996) Pollination compatibility of ‘Sundrop’ apricot and its progeny in the ‘Clutha’ series. N Z J Crop Hort Sci 24: 47-53.
14. McLaren GF, Fraser JA, Grant JE (1996) Some factors influencing fruit set in ‘Sunset’ apricot. N Z J Crop Hort Sci 24: 55-63.
15. Nyéki, J., Göndörné Pintér, M. & Szabó, Z. (1994). Recent data on fertilization of pear varieties. Acta Hort., 367, 87-91.
16. van den Eijnde J (1996) Pollination of pear by bumblebees (*Bombus terrestris* L.) and honeybees (*Apis mellifera* L.). Acta Hort 423: 73-78.
