# Supplementary material for: Valuing Insect Pollination Services with Cost of Replacement
Source: PLoS One. 2008 Sep 10;3(9):e3128. doi: 10.1371/journal.pone.0003128 (PMC2519790; doi:10.1371/journal.pone.0003128)
Supplement: Table S5 — Time required for one person to hand pollinate apple and pear fruit trees. (0.04 MB DOC) [file pone.0003128.s005.doc]

**Table S5.** Time required for one person to hand pollinate apple and pear fruit trees.

| Reference | Crop | Time required to pollinate 1 tree (minutes) | Time required to pollinate 1 hectare (man-days) |
| --- | --- | --- | --- |
| 1* | Apples | 45 | 154 |
| 2* | Apples | 60 | 206 |
| 3 | Apples | 60 | 206 |
| 4 | Apples | 60 | 206 |
| 5 | Apples | 60-90 | 206-309 |
| 6 | Apples | - | 12 |
| 7 | Pears | - | 41-164 |

The references indicated with an * are considered the most comprehensive and reliable, and yield mean time required of 180 man-days per hectare.

1. Snyder JC (1942) Commercial hand-pollination methods for apples in the north-west. Proc Am Soc Hort Sci 41: 183-186.
2. Overley FL, Bullock RM (1947) Pollen diluents and application of pollen to fruit trees. Proc Am Soc Hort Sci 49: 163-169.
3. Vansell GH, Griggs WH (1952) Honey bees as agents of pollination. U S Department of Agriculture Yearbook. pp. 88-107.
4. Griggs WH (1953) Pollination requirements of fruits and nuts. Calif Agric Exp Stn Circ 424: 4-35.
5. MacDaniels LH (1930) The possibilities of hand pollination in the orchard on a commercial scale. Proc Am Soc Hort Sci 27: 370-373.
6. Karmo EA, Vickery VR (1960) The fruit pollination of Nova Scotia. Gleanings in Bee Cult 88: 167-170, 187.
7. Ya T, Jia-sui X, Keming C (2003) Hand pollination of pears and its implications for biodiversity conservation and environmental protection -- A case study from Hanyuan county, Sichuan province, China. College of the Environment, Sichuan University Sichuan, China. Available: http://www.fao.org/Ag/AGP/AGPS/C-CAB/Castudies/pdf/6-005.pdf. Accessed 3 January 2007.
