# Supplementary material for: Valuing Insect Pollination Services with Cost of Replacement
Source: PLoS One. 2008 Sep 10;3(9):e3128. doi: 10.1371/journal.pone.0003128 (PMC2519790; doi:10.1371/journal.pone.0003128)
Supplement: Table S6 — Insect pollination replacement costs for the Western Cape deciduous fruit industry. (0.07 MB DOC) [file pone.0003128.s006.doc]

**Table S6**: Insect pollination replacement costs for the Western Cape deciduous fruit industry.

| Description | Apples | Apricots | Peaches & Nectarines | Pears | Plums & Prunes | Total |
| --- | --- | --- | --- | --- | --- | --- |
| Total production value derived from insect pollination using dependence factors* | 1265.4 | 38.9 | 147.6 | 696.2 | 133.3 | 2281.3 |
| Number of honeybee colonies to substitute all insect pollination (according to international stocking rates) | 37746 | 3603 | 7707 | 42572 | 20280 |  |
| Total hive rental cost (at ZAR261.60 per hive) to substitute all insect pollination* | 9.9 | 0.9 | 2.0 | 11.1 | 5.3 | 29.3 |
| Insect production value minus hive rental cost* | 1255.5 | 37.9 | 145.6 | 685.0 | 128.0 | 2252.0 |
| ***Replacement by dusting*** |  |  |  |  |  |  |
| Estimated production value with pollen dusting* | 194.5 | 6.0 | 22.7 | 107.0 | 20.5 | 350.6 |
| Total cost of pollen (at ZAR1597.38 per hectare) for dusting* | 30.1 | 5.8 | 12.3 | 17.0 | 5.4 | 70.6 |
| Total labour cost for dusting (at ZAR81.70 per day per hectare)* | 1.5 | 0.3 | 0.6 | 0.9 | 0.3 | 3.6 |
| Total insect pollination service value^†^ using dusting* | 1092.7 | 38.0 | 135.8 | 595.9 | 113.2 | 1975.6 |
| ***Replacement by hand pollination*** |  |  |  |  |  |  |
| Total production value for hand pollination (assuming comparable yield and quality to insect pollination)* | 1265.4 | 38.88 | 147.6 | 696.15 | 133.28 | 2281.3 |
| Total cost of pollen (at ZAR1185.48 per hectare) for hand pollination* | 22.4 | 4.3 | 9.1 | 12.6 | 4.0 | 52.4 |
| Percentage fruit set resulting from hand pollination | 50 | 25 | 25 | 6 | 25 |  |
| Number of fruit per tree resulting from insect pollination | 211 | 171 | 70 | 179 | 158 |  |
| Number of flowers that need to be pollinated per tree | 423 | 683 | 281 | 2976 | 634 |  |
| Number of flowers that need to be pollinated per hectare | 697183 | 853333 | 450000 | 4910072 | 903226 |  |
| Number of man days for hand pollination (at five seconds per flower) per hectare | 121 | 148 | 78 | 852 | 157 |  |
| Labour cost (at ZAR81.70 per day) per hectare (ZAR) | 9889 | 12104 | 6383 | 69645 | 12811 |  |
| Total labour cost for hand pollination, method 1* | 186.6 | 43.6 | 49.2 | 741.2 | 43.3 | 1064.0 |
| Total insect pollination service value^†^ using method 1* | 199.1 | 46.9 | 56.3 | 742.7 | 42.0 | 1087.1 |
| Labour cost for harvesting (ZAR per hectare) | 5670 | 15912 | 17025 | 4635 | 21657 |  |
| Labour cost for hand pollination (given insect dependence, fruit set and assuming hand pollination time is twice the harvest time) (ZAR per hectare) | 20412 | 61102 | 61290 | 140595 | 97023 |  |
| Total labour cost for hand pollination, method 2* | 385.2 | 220.2 | 472.4 | 1496.4 | 327.9 | 2902.0 |
| Total insect pollination service value^†^ using method 2* | 397.7 | 223.5 | 479.5 | 1497.8 | 326.6 | 2925.2 |
| Labour cost (at ZAR81.70/day) based on literature estimates for hand pollination (ZAR per hectare) | 14706 | 14706 | 14706 | 14706 | 14706 |  |
| Total labour cost for hand pollination, method 3* | 249.8 | 25.4 | 51.0 | 142.4 | 27.8 | 496.5 |
| Total insect pollination service value^†^ using method 3* | 262.3 | 28.8 | 58.1 | 143.9 | 26.5 | 519.6 |

* Value in ZAR millions; can be converted to US$ by dividing by 6.74388.

† Equivalent to income lost when opting for specified replacement method
