# Supplementary material for: Valuing Insect Pollination Services with Cost of Replacement
Source: PLoS One. 2008 Sep 10;3(9):e3128. doi: 10.1371/journal.pone.0003128 (PMC2519790; doi:10.1371/journal.pone.0003128)
Supplement: Table S7 — Managed honeybee replacement cost for the Western Cape deciduous fruit industry. Identical to Table S6, except for the use of observed instead of estimated number of honeybee colonies. (0.07 MB DOC) [file pone.0003128.s007.doc]

**Table S7.** Managed honeybee replacement cost for the Western Cape deciduous fruit industry.

| Description | Apples | Apricots | Peaches & Nectarines | Pears | Plums & Prunes | Total |
| --- | --- | --- | --- | --- | --- | --- |
| Proportion of total production value derived from insect pollination attributed to managed honeybees* | 528.4 | 0.3 | 0.6 | 145.3 | 133.3 | 807.9 |
| Number of managed honeybee colonies used in the Western Cape for deciduous fruit pollination^†^ | 15762 | 30 | 30 | 8888 | 21243 |  |
| Total hive rental cost (at ZAR261.60/hive)* | 4.1 | 0.008 | 0.008 | 2.3 | 5.6 | 12.0 |
| Managed honeybee production value minus hive rental cost* | 524.3 | 0.3^‡^ | 0.6^‡^ | 143.0 | 127.7 | 795.9 |
| ***Replacement by dusting*** |  |  |  |  |  |  |
| Estimated production value with pollen dusting* | 81.2 | - | - | 22.3 | 20.5 | 124.0 |
| Total cost of pollen (at ZAR1597.38 per hectare) for dusting* | 30.1 | - | - | 17.0 | 5.4 | 52.5 |
| Total labour cost for dusting (at ZAR81.70 per day per hectare)* | 1.5 | - | - | 0.9 | 0.3 | 2.7 |
| Total managed honeybee pollination service value^§^ using dusting* | 474.8 | - | - | 138.5 | 112.9 | 726.2 |
| ***Replacement by hand pollination*** |  |  |  |  |  |  |
| Total production value for hand pollination (assuming comparable yield and quality to managed honeybee pollination)* | 528.4 | - | - | 145.3 | 133.3 | 807.0 |
| Total cost of pollen (at ZAR1185.48 per hectare) for hand pollination* | 22.4 | - | - | 12.6 | 4.0 | 39.0 |
| Percentage fruit set resulting from hand pollination | 50 | - | - | 6 | 25 |  |
| Number of fruit per tree resulting from managed honeybee pollination | 88 | - | - | 37 | 158 |  |
| Number of flowers that need to be pollinated per tree | 176 | - | - | 621 | 634 |  |
| Number of flowers that need to be pollinated per hectare | 291130 | - | - | 1025104 | 903226 |  |
| Number of man days for hand pollination (at five seconds per flower) per hectare | 51 | - | - | 178 | 157 |  |
| Labour cost (at ZAR81.70 per day) per hectare (ZAR) | 4129 | - | - | 14540 | 12811 |  |
| Total labour cost for hand pollination, method 1* | 77.9 | - | - | 154.8 | 43.3 | 276.0 |
| Total managed honeybee pollination service value^§^ with method 1* | 96.2 | - | - | 165.0 | 41.8 | 303.0 |
| Labour cost for harvesting (ZAR per hectare) | 5670 | - | - | 4635 | 21657 |  |
| Labour cost for hand pollination (given managed honeybee pollinated fruit, fruit set and assuming hand pollination time is twice the harvest time) (ZAR per hectare) | 8524 | - | - | 29353 | 97023 |  |
| Total labour cost for hand pollination, method 2* | 160.9 | - | - | 312.4 | 327.9 | 801.2 |
| Total managed honeybee pollination service value^§^ with method 2* | 179.1 | - | - | 322.7 | 326.4 | 828.2 |
| Labour costs (at ZAR81.70/day) based on literature estimates for hand pollination (ZAR per hectare) | 14706 | - | - | 14706 | 14706 |  |
| Total labour cost for hand pollination, method 3* | 104.3 | - | - | 29.7 | 27.8 | 161.9 |
| Total managed honeybee pollination service value^§^ with method 3* | 122.6 | - | - | 40.0 | 26.3 | 188.9 |

Identical to Table S6, except for the use of observed instead of estimated number of honeybee colonies.

* Value in ZAR millions; can be converted to US$ by dividing by 6.74388.

† Personal communications from the South African Bee Industry Organisation and Deciduous Fruit Producers’ Trust

‡ Observed contribution of managed honey bees to pollination is less than one percent and was consequently omitted from replacement calculations.

§ Equivalent to income lost when opting for specified replacement method.
